# The Activity of Protectin DX, 17 HDHA and Leukotriene B4 Is Correlated with Interleukin-1β (IL-1β) and Interleukin-1 Receptor Antagonist (IL-1Ra) in the Early Subacute Phase of Stroke

**DOI:** 10.3390/ijms26189088

**Published:** 2025-09-18

**Authors:** Dariusz Kotlega, Arleta Drozd, Agnieszka Zembron-Lacny, Barbara Morawin, Karina Ryterska, Malgorzata Szczuko

**Affiliations:** 1 Department of Pharmacology and Toxicology, University of Zielona Gora, 65-417 Zielona Gora, Poland; 2 Northampton General Hospital, Northampton NN1 5BD, UK; 3 Department of Applied Microbiology and Human Nutrition Physiology, Faculty of Food Sciences and Fisheries, West Pomeranian University of Technology, 70-310 Szczecin, Poland; 4 Faculty of Medicine, University of Warsaw, 02-089 Warsaw, Poland; 5 Department of Applied and Clinical Physiology, University of Zielona Gora, 65-417 Zielona Gora, Poland; 6 Department of Human Nutrition and Metabolomics, Pomeranian Medical University in Szczecin, 71-460 Szczecin, Poland; 7 Department of Bromatology and Nutritional Diagnostics, Pomeranian Medical University in Szczecin, 71-460 Szczecin, Poland; malgorzata.szczuko@pum.edu.pl

**Keywords:** ischaemic stroke, interleukin-1 beta (IL-1β), interleukin-1 receptor antagonist (IL-1Ra), inflammation, eicosanoids, neuroinflammation

## Abstract

Ischemic stroke is a leading cause of mortality and disability in adults. The inflammatory cascade is driven by various inflammatory molecules, such as interleukin-1β (IL-1β), and counteracted by its antagonist, interleukin-1 receptor antagonist (IL-1Ra). Eicosanoids are inflammatory derivatives of free fatty acids. Arachidonic acid (AA) derivatives exhibit pro-inflammatory activity, while eicosapentaenoic acid (EPA) and docosahexaenoic acid (DHA) derivatives, known as specialized pro-resolving mediators, have anti-inflammatory properties. This study aimed to analyze potential associations between eicosanoids and key inflammatory molecules, including IL-1β and its antagonist IL-1Ra. In this prospective study, we investigated inflammatory molecules in 73 ischemic stroke patients. We analyzed interactions between IL-1β, IL-1Ra, and eicosanoids as follows: resolvin E1, prostaglandin E2, resolvin D1, lipoxin A4 (5S, 6R, 15R), protectin DX, maresin 1, leukotriene B4, 18RS-HEPE, 13S-HODE, 9S-HODE, 15S-HETE, 17 HDHA, 12S-HETE, 5-oxo-ETE, and 5-HETE. In 73 ischemic stroke patients, mean IL-1β was 1.31 ± 1.54 pg/mL and IL-1Ra 810.8 ± 691.0 pg/mL. Spearman correlations showed positive associations between IL-1β and protectin DX (ρ = 0.56, *p* < 0.001), and 17 HDHA (ρ = 0.26, *p* < 0.05) and 5-oxo-ETE (ρ = 0.27, *p* < 0.05). IL-1Ra correlated negatively with protectin DX (ρ = −0.58, *p* < 0.001) and 17 HDHA (ρ = −0.29, *p* < 0.05), and positively with leukotriene B4 (ρ = 0.34, *p* < 0.005). After multivariable adjustment, associations with IL-1β lost statistical significance, whereas the inverse relationships between IL-1Ra and protectin DX/17 HDHA remained significant (*p* < 0.005). Despite the known anti-inflammatory roles of protectin DX and 17 HDHA, and the pro-inflammatory role of leukotriene B4, their activity in the early subacute phase of ischemic stroke appears to be influenced by complex interplays, possibly mediated by IL-1β and IL-1Ra. The activity of protectin DX, 17 HDHA, and leukotriene B4 is correlated with IL-1β and IL-1Ra levels in the early subacute phase of stroke.

## 1. Introduction

Ischemic stroke is a leading cause of mortality and disability in adults. According to prevalence estimates, ischemic stroke morbidity is projected to increase from 3.9% to 6.4% by 2050 [1]. The primary cause of ischemic stroke is atherosclerosis, which underlies the atherothrombotic pathomechanism of stroke. The development and progression of atherosclerosis are largely driven by chronic inflammation. In the acute phase of stroke, inflammation rapidly intensifies due to arterial obstruction and ischemia, triggering an inflammatory cascade. As this process unfolds, a subsequent shift occurs toward the resolution of inflammation in the later phase of stroke [2].

The inflammatory cascade is mediated by multiple inflammatory molecules, prothrombotic activity, and blood–brain barrier (BBB) disruption. Initially, platelet activation occurs, and leukocytes bind to P-selectin. This process is modulated by adhesion molecules such as vascular cell adhesion molecule-1 (VCAM-1), E-selectin, and intercellular adhesion molecule-1 (ICAM-1). The inflammatory response includes increased expression of serum interleukins, such as IL-1β, which stimulates the secretion of TNF-α, IFN-γ, matrix metalloproteinases (MMPs), and IL-6 [3,4].

Interleukin-1 beta (IL-1β) plays a crucial role in the acute phase of stroke within the inflammatory cascade and serves as a long-term chronic contributor to atherosclerosis development and increased stroke risk. Interleukin-1 exists in two forms, IL-1α and IL-1β, which are activated together. Both exert strong pro-inflammatory effects and are primarily synthesized by microglia. IL-1β is released by leukocytes, while IL-1α is activated by platelets. The activation of IL-1β requires nuclear factor κ-light-chain-enhancer of activated B cells (NF-κB) and is mediated by pro-inflammatory stimuli that engage Toll-like receptors (TLRs) [5,6]. IL-1β exerts both pro-inflammatory and procoagulant effects by inducing the production of MMP-9, IL-6, IFN-γ, TNF-α, and various chemokines. It promotes vascular smooth muscle growth and the activation of cell adhesion molecules, facilitating leukocyte and monocyte adhesion to the endothelium [7,8,9]. IL-1β plays a key role in both neuroinflammation within the brain and systemic inflammatory responses [10]. Chronic elevation in IL-1β, as seen in metabolic syndrome and type 2 diabetes, may predispose individuals to more severe strokes [11]. Higher IL-1β levels are associated with an increased risk of cardiovascular events, as indicated by the Framingham CVD risk score, and with smoking status in diabetic patients [12]. Canakinumab, a monoclonal antibody targeting IL-1β, has been shown to reduce the risk of myocardial infarction, cardiovascular death, and stroke in patients with coronary heart disease (CHD). This effect was accompanied by a decrease in C-reactive protein (CRP) levels, though no significant changes in lipid profiles were observed [7,13].

Interleukin-1 receptor antagonist (IL-1Ra) exerts anti-inflammatory effects by inhibiting both IL-1α and IL-1β. The recombinant form of IL-1Ra, commercially known as anakinra, has been approved for the treatment of autoimmune conditions such as rheumatoid arthritis, Still’s disease, cryopyrin-associated periodic syndrome (CAPS), familial Mediterranean fever (FMF), and periodic fever syndromes [14,15]. Studies have shown that anakinra reduces inflammation following stroke and decreases infarct volume by 38% in animal models of stroke. Additionally, it improves clinical outcomes in stroke patients while maintaining a favourable safety profile [16]. However, in patients with ST-elevation myocardial infarction (STEMI), anakinra did not significantly impact the risk of recurrent myocardial infarction (MI), stroke, unstable angina, or symptomatic heart failure [17].

Eicosanoids are inflammatory derivatives of free fatty acids (FFAs). Initially, they were primarily considered pro-inflammatory oxygenated derivatives of arachidonic acid (AA), but some also exhibit anti-inflammatory properties. Leukotriene B4 (LTB4) is a chemotactic eicosanoid derived from the lipoxygenase (LOX) pathway, while prostaglandins, such as prostaglandin E2 (PGE2), are cyclooxygenase (COX)-derived molecules synthesized in macrophages [18]. Anti-inflammatory derivatives of docosahexaenoic acid (DHA) and eicosapentaenoic acid (EPA), including maresins, resolvins, and protectins, are collectively known as specialized pro-resolving mediators (SPMs) [19,20]. Another class of SPMs includes lipoxins, which are synthesized from AA [21]. Arachidonic acid, the primary long-chain omega-6 polyunsaturated fatty acid (PUFA) involved in eicosanoid synthesis, is positively associated with low-density lipoprotein cholesterol (LDL-C) and inversely associated with IL-1Ra, IL-1, and triglyceride levels [22].

The aim of this study was to analyze potential associations between eicosanoids and key inflammatory molecules, including IL-1β and its antagonist IL-1Ra, in the early subacute phase of stroke. These interactions may play a role in stroke patients, both in the context of chronic inflammation as a risk factor for atherosclerosis and in the inflammatory cascade during the acute and subacute phases of stroke. To our knowledge, this is the first study to investigate these associations, especially in the timing when pro-inflammatory activity is pronounced and the pro-resolving molecules are on the early phase of production. Such analyses have not been conducted previously. Understanding these potential interactions may provide valuable insights for future research on neuroinflammation and ischemic stroke risk.

## 2. Results

The demographic characteristics of the cohort are presented in Table 1.

The mean value and SD for IL-1β (pg/mL) was 1.31 ± 1.54 and 810.78 ± 691.02 for IL1-Ra (pg/mL).

Unadjusted correlations between eicosanoids and IL-1β are presented in Table 2.

**Figure 1:**
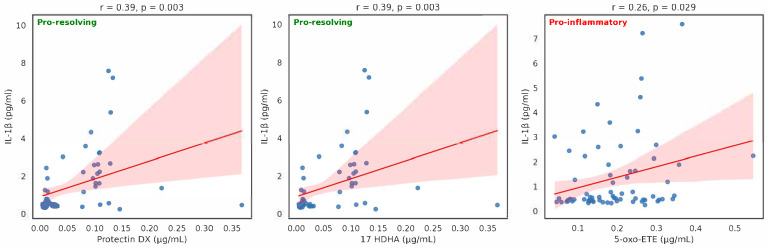
Correlations between eicosanoids and IL-1β (Spearman ρ and unadjusted *p*-values).

We observed significant positive correlations between IL-1β and protectin DX, 17 HDHA, and 5-oxo-ETE (Figure 1) with Spearman rho, bootstrap 95% CI: 0.331, 0.745; 0.042, 0.464 and 0.026, 0.480, respectively. Protectin DX was still significant after Bonferroni correction (*p* < 0.001) and FDR correction (adjusted *p* < 0.05). After multivariate analysis for confounding factors, none of these eicosanoids remained statistically significant. We additionally performed PCA extracting five components explaining 45.3% of variance. The most significant components are as follows:

- PC1 (23.4% variance)—eicosanoid metabolic network, top loadings: 15S HETE (0.377), Maresin 1 (0.353), 13S HODE (0.328), 5 HETE (0.322).
- PC2 (12.5% variance) lipid-inflammatory axis—non-HDL (0.559), LDL (0.468), TG (0.435), CRP (0.356).
- PC3 (9.4% variance)—IL-1β resolution axis: IL-1β (0.549), Protectin DX (0.426), Resolvin E1 (0.349).

Unadjusted correlations between eicosanoids and IL1-Ra are presented in Table 3.

**Figure 2:**
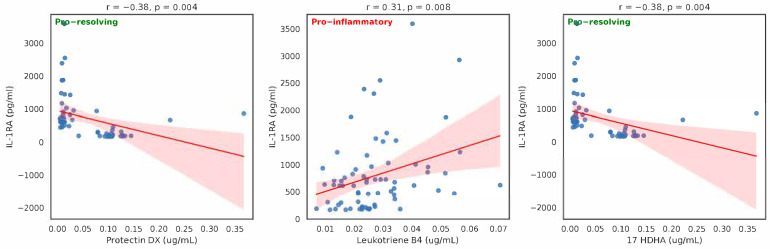
Correlations between eicosanoids and IL1-Ra (Spearman ρ and unadjusted *p*-values).

We observed statistically significant inverse correlations between IL1-Ra, protectin DX and 17 HDHA, and positive correlation with leukotriene B4 (Figure 2). Their Spearman rho, bootstrap 95% CI were as follows: −0.713, −0.380; −0475, −0.073 and 0.125, 0.528, respectively. Protectin DX maintains significance even after the Bonferroni correction. The multivariate analysis showed that after adjusting for age, sex, BMI, CRP, and lipids, protectin DX and 17 HDHA remained statistically significant (*p*-value < 0.005).

Three major components of PCA for IL1-Ra and eicosanoids explain the 44.8% of variance. The most significant components are as follows:

- PC1 (23.5% variance)—eicosanoid metabolic network, top loadings: 15S HETE (0.378), Maresin 1 (0.354), 13S HODE (0.328), 5 HETE (0.32).
- PC2 (12.5% variance) lipid-inflammatory axis—non-HDL (0.559), LDL (0.467), TG (0.435), CRP (0.355).
- PC3 (8.8% variance)—IL1-Ra primary axis, top loadings: IL1-Ra (0.546), Protectin DX (−0.528), Resolvin E1 (−0.282).

**Figure 3:**
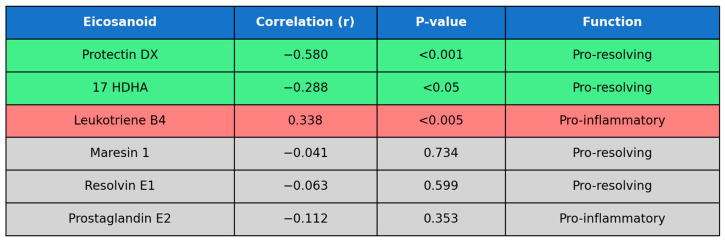
Summary of unadjusted correlations between eicosanoids and IL1-Ra and their biological functions. Green—statistically significant pro-resolving mediators, red—statistically significant pro-inflammatory mediator, grey—statistically non-significant.

The summary of unadjusted correlations between IL1-Ra and eicosanoids are presented in Figure 3.

## 3. Discussion

We observed significant positive correlations between the pro-inflammatory IL-1β and protectin DX, and 17 HDHA and 5-oxo-ETE (Figure 1). The anti-inflammatory IL-1Ra was inversely associated with the first two eicosanoids, i.e., protectin DX and 17 HDHA, while a positive correlation was found with leukotriene B4 (Figure 2). In view of the antagonist activities of IL-1β and IL-1Ra, the opposite correlations of protectin DX and 17 HDHA are consistent.

17 HDHA (hydroxy-docosahexaenoic acid, 17RS HDHA) serves as a precursor for the D-series of resolvins and comprises both 17-S and 17-R enantiomers. It belongs to the group of n-3 free fatty acid derivatives known as specialized pro-resolving mediators (SPMs). These molecules function to inhibit cytokine production and promote the resolution of inflammation by reducing the synthesis of cytokines such as IL-10, TNFα, and IL-12. Docosahexaenoic acid (DHA) is the precursor for D-series resolvins, while eicosapentaenoic acid (EPA) gives rise to E-series resolvins. DHA acts as a substrate for 15-LOX, and 17 HDHA is an intermediate produced prior to the generation of D-series resolvins following the involvement of 5-LOX. Studies have demonstrated that 17 HDHA exerts anti-inflammatory activity by enhancing human B cell production [23,24]. It has also been negatively correlated with TNF-α, IL-6, MCP-1, fasting glucose, and reduced gene expression of NF-κB in diet-induced obese animals [25]. Levels of 17 HDHA are lower in stroke patients compared to healthy controls [26]. Our findings, in which 17 HDHA positively correlates with IL-1β and negatively with IL-1Ra, might suggest a potential pro-inflammatory role. In contrast to our results, other studies indicate an anti-inflammatory role for 17 HDHA, with negative correlations observed with IL-1β and other cytokines and adhesion molecules such as TNF-α, MIP-2, CXCL1/KC, VCAM-1, ICAM-1, and LFA-1 in an animal model of colitis. These effects are primarily a ttributed to the increased conversion of 17 HDHA to resolvins and their subsequent anti-inflammatory actions [27].

Similar to the correlation observed for 17 HDHA, we found that protectin DX (PDX) positively correlated with IL-1β and negatively with IL-1Ra. Protectins belong to the family of specialized pro-resolving mediators (SPMs) [28]. Levels of protectin DX are not different in stroke patients compared to healthy controls [26]. PDX, an isomer of protectin D1, is synthesized from DHA through dual lipoxygenation mediated by 8-LOX and 15-LOX. It exhibits both acute and chronic anti-inflammatory activities by inhibiting the AMPK/NF-κB pathway and modulating PPARγ transcriptional activity. PDX plays a role in limiting insulin resistance, arthritis, and end-stage renal failure. Additionally, it possesses antioxidant, antiviral, and anti-fibroproliferative properties, preserves tissue functionality, enhances macrophage phagocytosis, and inhibits both the NLRP3 inflammasome pathway and transforming growth factor-β1 (TGF-β1). Moreover, it improves insulin sensitivity, inhibits platelet aggregation, and reduces mortality in an animal model of sepsis [29,30,31,32]. Levels of protectin D1 vary depending on the condition. It is increased in infection and sepsis, yet decreased in obesity, DHA-treated human neuroblastoma cells, and multiple sclerosis. A similar pattern may apply to PDX, although this has not been studied yet. Only a limited number of studies have examined these eicosanoids in stroke patients. Some reports indicate that the levels of PDX and leukotriene B4 do not differ between stroke patients and controls, whereas levels of 17 HDHA and 5-oxo-ETE are lower in stroke patients compared to controls. Other studies have shown that leukotriene B4 is increased in stroke patients and correlates positively with poor clinical outcomes in stroke survivors. Furthermore, depending on the LTB4 haplotype, the risk of stroke may be enhanced by up to 2.3-fold [26,32,33,34,35].

Our findings regarding the correlations of 17 HDHA and protectin DX may be explained by the activation of compensatory pro-resolving mechanisms. These observations do not necessarily imply a pro-inflammatory role for 17 HDHA and PDX, particularly as our study was conducted during the early subacute phase of stroke. In this phase, IL-1β initiates the inflammatory cascade, and in response, specialized pro-resolving mediators (SPMs) are activated to mitigate inflammation. Specifically, IL-1β stimulates 15-LOX, leading to increased synthesis of 17 HDHA and protectin DX. At this early stage, the conversion of 17 HDHA into D-series resolvins might be less efficient due to an overload of 15-LOX, and the synthesis of 17 HDHA could be delayed since the resolving phase typically follows the initial inflammatory cascade. Furthermore, enhanced production of SPMs may reduce the need for IL-1Ra synthesis, as pro-resolving molecules might limit its production. Alternatively, the kinetics of activation may play a role, with SPMs being activated before IL-1Ra, thereby resulting in an inverse correlation with IL-1Ra. Ultimately, our findings may reflect the homeostatic balance between pro- and anti-inflammatory mediators during the early subacute phase of stroke. Other explanations are also possible. Higher SPM levels might be a consequence, not a cause, and they could rise because an upstream inflammatory trigger is not being resolved. The same enzymes (for example 15-LOX or PLA2) might increase both SPM precursors and cytokines. Alternatively, SPM receptors or downstream signaling could be impaired, so higher SPMs have less effect. Distinguishing these possibilities requires mechanistic studies and repeated sampling over time.

We observed a positive correlation between leukotriene B4 (LTB4) and IL-1Ra. LTB4 is an eicosanoid within the leukotriene family that plays a critical role in mediating inflammatory responses. Levels of LTB4 are not different in stroke patients compared to healthy controls [26]. It is synthesized from arachidonic acid (AA) via the 5-lipoxygenase (5-LOX) pathway, with its signaling mediated through the BLT1 and BLT2 receptors. LTB4 activates Toll-like receptors (TLRs), nuclear factor κB (NF-κB), and mitogen-activated protein kinases (MAPKs), promoting neutrophil activation, phagocytosis, chemotaxis, and the recruitment of CD4+ and CD8+ T cells, as well as Th1 and Th2 cells. It further enhances the production of cytokines including IL-2, IL-5, IL-6, IL-8, and MCP-1, and increases the synthesis of IL-1 (α/β) [33]. A bilateral interaction exists between IL-1β and LTB4: blocking BLT1 or BLT2 suppresses the stimulation of the NLRP3 inflammasome and IL-1β production, whereas inhibition of the IL-1β receptor using IL-1Ra reduces LTB4 synthesis [36,37]. Our finding that IL-1Ra positively correlates with pro-inflammatory LTB4 suggests an imbalance of inflammatory mediators during the early subacute phase of stroke. In this phase, elevated LTB4 levels may activate IL-1Ra to initiate pro-resolving mechanisms. Additionally, IL-1Ra may be activated by other pro-inflammatory molecules beyond LTB4.

We also detected a positive correlation between IL-1β and 5-oxo-ETE (5-oxo-6,8,11,14-eicosatetraenoic acid). This eicosanoid is a pro-inflammatory mediator synthesized via the oxidation of 5-HETE, which is generated by the 5-LOX-mediated metabolism of arachidonic acid [38]. 5-oxo-ETE promotes the migration of neutrophils, eosinophils, monocytes, and basophils through its receptor, OXE-R, which can be inhibited by EPA. In an animal model of myocardial infarction, 5-oxo-ETE was shown to exacerbate apoptosis and ischemic injury [39]. Although its role in stroke remains poorly understood, previous studies have demonstrated that levels of 5-oxo-ETE are lower in stroke patients compared to healthy controls [26].

Ischemic stroke results from an interruption in cerebral blood flow, which leads to neuronal damage, inflammation, and oxidative stress. Inflammation plays a central role in the pathophysiology of ischemic stroke, with IL-1β recognized as a critical mediator. IL-1β triggers the production of eicosanoids, bioactive lipid mediators derived from arachidonic acid that exert both pro-inflammatory and prothrombotic effects. The IL-1β–eicosanoid axis facilitates inflammatory signaling and cytokine infiltration into the brain, while also promoting mitochondrial dysfunction, excitotoxicity, and oxidative stress. Understanding the link between IL-1β and eicosanoids provides insights into how inflammation affects ischemic injury and may help inform preventive strategies.

In our study, we demonstrated that IL-1β positively correlates with several eicosanoids (protectin DX, 17 HDHA, and 5-oxo-ETE), whereas IL-1Ra was inversely associated with protectin DX and 17 HDHA, and positively correlated with LTB4. Several pathogenetic links may explain these interactions. First, IL-1β strongly induces cyclooxygenase-2 (COX-2), which converts arachidonic acid into pro-inflammatory eicosanoids such as prostaglandins and thromboxanes [40]. Thromboxane A2 (TXA2) induces vasoconstriction and platelet aggregation, potentially worsening microvascular obstruction in the brain. Studies indicate that inhibitors of COX-2 and 5-LOX can reduce IL-1β-mediated eicosanoid synthesis [41]. Furthermore, adiponectin and IL-1β synergistically increase the production of PGE2, a crucial pro-inflammatory eicosanoid [42]. Although PGE_2_ did not show statistically significant associations in our analyses, it remains mechanistically important in post-stroke inflammation and its levels are increased in stroke. PGE_2_ is a central product of COX-mediated arachidonic acid metabolism and has well-established roles in vascular tone, leukocyte recruitment, and blood–brain barrier integrity. It can exert both pro-inflammatory and regulatory effects depending on receptor subtype and cellular context. The lack of significance in our study may reflect small sample size, timing of sampling, or inter-individual variability. Future studies with larger cohorts and serial sampling should further examine the contribution of PGE_2_ [43,44].

A second factor may be the activation of phospholipase A2 (PLA2), which is also induced by IL-1β. PLA2 increases the release of arachidonic acid from membrane phospholipids, providing the substrate for both cyclooxygenase and lipoxygenase pathways. The lipoxygenase pathway leads to the production of leukotrienes, including LTB4, which is responsible for leukocyte recruitment and the inflammatory response in ischemic stroke [34]. Additionally, IL-1β is known to weaken the blood–brain barrier (BBB), which, in combination with leukotrienes and prostaglandins, exacerbates the inflammatory process within the ischemic brain region [45]. IL-1β has also been shown to activate PGE2 in synoviocytes in rheumatoid arthritis [46]. Elevated levels of certain eicosanoids, such as PGE2, can further upregulate IL-1β synthesis, potentially creating a pro-inflammatory loop. Conversely, lipoxins, as pro-resolving molecules, may be upregulated in an IL-1β-dependent manner and contribute to the resolution of inflammation. Further elucidation of the pathogenetic interplay between IL-1β and eicosanoids may contribute to the development of neuroprotective and anti-inflammatory interventions in stroke patients, potentially involving the use of agents such as the IL-1β antagonist anakinra, COX-2 inhibitors, or leukotriene receptor antagonists.

Interleukin-1 receptor antagonist (IL-1Ra) is an endogenous anti-inflammatory cytokine that competes with interleukin-1 (IL-1) for receptor binding, thereby inhibiting IL-1–mediated inflammatory responses. Several potential links may exist between IL-1Ra and eicosanoids in the pathogenesis of ischemic stroke. IL-1Ra counterbalances IL-1β as a potent anti-inflammatory protein and is synthesized after stroke to promote pro-resolving activity and limit ischemia-induced damage. A key common factor is its influence on the activation of Toll-like receptor 4 (TLR4) on immune cells, which subsequently activates NF-κB and promotes the synthesis of pro-inflammatory cytokines such as IL-1β. These pathways have been discussed above in relation to the metabolic activities of certain eicosanoids. Antagonizing IL-1β with IL-1Ra can facilitate anti-inflammatory reactions. IL-1Ra may be beneficial as an anti-inflammatory molecule by inhibiting oxidative stress and prothrombotic activity. By blocking IL-1β, it also prevents the infiltration of cytokines into the brain parenchyma through the blood–brain barrier (BBB). Exogenous IL-1Ra (anakinra) has been shown to reduce infarct size in stroke models [47]; however, anakinra was found to be ineffective in reducing brain edema in patients with intracerebral hemorrhage, and further studies are planned [48,49]. Another link between IL-1β, IL-1Ra, and eicosanoids is that IL-1Ra blocks IL-1β and subsequently inhibits IL-1β–induced synthesis of cyclooxygenase-2 (COX-2), the enzyme responsible for the production of pro-inflammatory prostaglandins. COX activity also upregulates the synthesis of thromboxane A2 (TXA2), which promotes vasoconstriction and platelet aggregation; thus, the effect of IL-1Ra on COX results in anti-inflammatory and antithrombotic activity. Additionally, IL-1Ra inhibits the activation of cytosolic phospholipase A2 (cPLA2), which cleaves fatty acids such as arachidonic acid (AA) from membrane phospholipids, thereby reducing the substrate available for eicosanoid synthesis. Moreover, IL-1Ra significantly inhibits IL-1–induced PGE2 production, suggesting a potential indirect effect on the enzymes involved in PGE2 synthesis, possibly including microsomal prostaglandin E synthase-1 (mPGES-1), which converts prostaglandin H2 (PGH2) to pro-inflammatory prostaglandin E2 (PGE2) [50]. Furthermore, IL-1Ra is indirectly involved in anti-inflammatory activity by inhibiting IL-1β and downregulating 5-lipoxygenase (5-LOX), which normally leads to the synthesis of pro-inflammatory leukotrienes such as LTB4. By inhibiting the production of PGE2 and leukotrienes, IL-1Ra reduces the risk of BBB disruption, preventing the infiltration of inflammatory molecules and edema formation. Finally, as a result of its anti-inflammatory activities, IL-1Ra enables pro-resolving eicosanoids to initiate the resolution of the inflammatory process.

Principal Component Analysis of IL1-beta and IL1-RA with eicosanoids revealed that both inflammatory markers demonstrate their strongest loadings on the same resolution axis (PC3), but in inverse relationship to specialized pro-resolving mediators. IL1-beta (loading: +0.521) and IL1-RA (loading: +0.546) both oppose Protectin DX (loadings: −0.485 and −0.528, respectively) and Resolvin E1 (loadings: −0.301 and −0.282, respectively) on this axis, with direct correlations confirming these inverse relationships (IL1-beta vs. Protectin DX: r = −0.341; IL1-RA vs. Protectin DX: r = −0.377). This parallel positioning suggests that both pro-inflammatory IL1-beta and anti-inflammatory IL1-RA represent active inflammatory states distinct from the resolution phase, which is characterized by specialized pro-resolving mediator dominance.

The main limitation of our study is that it was conducted during the early subacute phase of ischemic stroke, a period characterized by the initiation of the inflammatory cascade, during which certain processes, like the resolution of inflammation, may not be fully established yet. Blood samples were collected on day 7 after symptom onset to capture the early subacute/transitional inflammatory phase following the initial ischemic insult. We recognize that peak IL-1β often occurs earlier (within 24–72 h) and that day-7 sampling may reflect the overlap between pro-inflammatory activation and initiation of resolution pathways. The timing of blood sample collection could reflect overlapping of pro- and anti-inflammatory processes in the dynamic inflammatory stroke responses in a transitional stage between acute and subacute inflammation. This timing might have influenced the observed associations. Moreover, by day 7, BBB disruption may persist in some patients but tend to be variable. Peripheral plasma eicosanoid levels provide an accessible surrogate signal but may not fully mirror intraparenchymal changes due to BBB status. Thus, interpretations about central neuroinflammation should be cautious.

Additionally, our analysis is based on a single time-point measurement of inflammatory parameters. Cytokine and eicosanoid levels are dynamic: early peaks of IL-1β within the first 24–72 h may have diminished by day 7, and IL-1Ra levels may be rising as a compensatory response. Serial sampling would be required to determine temporal kinetics and causality; we recommend this for future studies. Given that both activation and resolution of inflammation are dynamic continuous processes, our findings should be interpreted with caution.

Both embolic and atherothrombotic stroke mechanisms were included. Mechanism-specific inflammatory responses may differ, which might have affected our results. There was no breakdown analysis of stroke mechanism subgroups, because our sample size limits robust subgroup inference. The type and dose of statins were not recorded and analyzed, although local practice is such that most of the patients are commenced on atorvastatin. This limits the results, because statins can affect eicosanoids synthesis.

Ischemic stroke results from a reduction in cerebral blood flow, triggering a complex metabolic and inflammatory cascade. Identifying therapeutic agents that modulate inflammation and thrombotic processes may be clinically relevant for reducing ischemic injury and improving stroke outcomes. Targeting the IL-1β–IL-1Ra–eicosanoids axis, for example, through agents such as anakinra, may offer potential benefits in controlling inflammation and promoting neuroprotection in stroke survivors. Interestingly, our study identified associations between IL-1β, IL-1Ra, and certain eicosanoids that do not fully align with their established pro- or anti-inflammatory roles. This discrepancy may be explained by the evolving inflammatory response, complex metabolic feedback loops, or the transient nature of cytokine and eicosanoid fluctuations in the early subacute phase of stroke. Further longitudinal studies with serial measurements are warranted to elucidate these interactions and their potential therapeutic implications.

## 4. Material and Methods

### 4.1. Subjects

A prospective investigation enrolled 73 individuals diagnosed with ischemic stroke within the 24 h since the onset of the symptoms. Patients were selected based on confirmed stroke established through clinical assessment and supplementary tests, including CT or MRI imaging. Information regarding comorbidities and routine blood tests were recorded, including lipid profile and CRP. The cohort included cases with either embolic or atherothrombotic mechanisms and the distribution of subgroups according to the TOAST classification was as follows: large-artery atherosclerosis (n = 30), cardioembolism (n = 9), small vessel occlusion/lacunar (n = 20), other determined causes (n = 0), and undetermined cause (n = 14) [51]. For this study, ischemic stroke was defined as the abrupt onset of either localized or diffuse cerebral dysfunction lasting at least 24 h or verified by imaging. Patients were excluded if imaging revealed intracranial hemorrhage, if they showed signs of active infection (e.g., body temperature above 37.4 °C), if clinical or laboratory findings indicated infection, or if they had active autoimmune diseases, malignancies, or disturbances in speech or consciousness resulting from cerebral, metabolic, or other conditions that could compromise neuropsychological assessments. All subjects were admitted to the Neurology Department of a regional hospital in Poland and were of Caucasian descent. None had taken omega-3 supplements prior to hospitalization. During their stay, they were treated with statins and acetylsalicylic acid. Statin type and dose were not recorded and did not enter as covariates. Statin and acetylsalicylic acid therapies were continued or initiated on admission. AI-assisted copy editing was used only for improvements to human-generated texts for readability and style, and to ensure that the texts are free of errors in grammar, spelling, punctuation, and tone. The authors reviewed and edited the content as needed and take full responsibility for the content of the published article. The study protocol received approval from the Bioethics Committee at the Regional Medical Chamber in Zielona Gora, Poland (decision no. 08/73/2017, the 28 February 2017) and adhered to the principles of the Declaration of Helsinki. Informed consent was obtained from all individual participants included in the study.

### 4.2. Eicosanoids Analysis

Venous blood samples were collected on the seventh day after symptom onset (n = 73). Blood was collected in the morning, fasting samples, processed on the day of collection, were centrifuged, and plasma aliquots were transferred to −80 °C for long-term storage as soon as logistically possible. Where immediate −80 °C storage was not available, aliquots were held at −20 °C for a short period (<48 h) prior to transfer to −80 °C. High-performance liquid chromatography (HPLC) was performed using methanol and acetic acid as mobile phases, along with double-distilled water and buffers, as previously described. Prior to analysis, samples were filtered through 0.22 µm nylon filters. The study examined various inflammatory mediators, including resolvin D1, maresin 1, prostaglandin B2, 5(S),6(R),15(R)-lipoxin A4, 5(S),6(R)-lipoxin A4, leukotriene B4, 16(RS)-HEPE, 5(S)-HETE, 12(S)-HETE, 15(S)-HETE, 5(S)-oxo-ETE, 10(S),17(R)-DiDHA, 16(R)/16(S)-HETE, 9(S)-HODE, 13(S)-HODE, and 17(RS)-HDHA. These derivatives were extracted from 0.5 mL of plasma using solid-phase extraction with RP-18 SPE columns (Agilent Technologies, Santa Clara, CA, USA).

HPLC separations were carried out on an Agilent 1260 liquid chromatograph, equipped with a degasser (model G1379B), bin pump (model G1312B), column oven (model G1316A), and DAD detector (model G1315C VL+). Sample injection was performed using model G1329B. Data acquisition, instrument control, and analysis were managed with Agilent ChemStation software (Agilent Technologies, Cheadle, UK). The column oven temperature was maintained at 21 °C. Separation was achieved using a Thermo Scientific Hypersil BDS C18 column (100 mm × 4.6 mm, 2.4 µm). A gradient method was applied, utilizing solvent mixtures: phase A (methanol/water/acetic acid, 50/50/0.1, *v*/*v*/*v*) and phase B (methanol/water/acetic acid, 100/0/0.1, *v*/*v*/*v*). The percentage of buffer B in the mobile phase started at 30% (0.0–2.00 min), increased to 80% at 33 min, reached 98% between 33.1 and 37.5 min, and returned to 30% from 40.3 to 45 min. The flow rate was maintained at 1.0 mL/min, with an injection volume of 60 μL.

The DAD detector monitored peak absorption at different wavelengths: 235 nm for 16(RS)-HEPE, 17(RS)-HDHA, 9(S)-HODE, 13(S)-HODE, 5(S)-HETE, 12(S)-HETE, and 15(S)-HETE; 280 nm for prostaglandin B2 (internal standard), 5(S)-oxo-ETE, resolvin E1, 10(S),17(R)-DiDHA, maresin 1, and leukotriene B4; 210 nm for prostaglandin E2, 16(R)-HETE, and 16(S)-HETE (which co-eluted as a single peak); and 302 nm for 5(S),6(R)-lipoxin A4, 5(S),6(R),15(R)-lipoxin A4, and resolvin D1. Analyte identification was confirmed through peak absorbance spectra analysis. Quantification was based on peak areas using internal standard calibration, with ChemStation Software (Agilent Technologies, Cheadle, UK) used for data processing [26,52].

### 4.3. Interleukin-1 Beta and Interleukin-1 Receptor Antagonist Analysis

Cytokines IL-1β and IL-1Ra concentrations were determined using ELISA kits from SunRed Biotechnology Company (Shanghai, China) with detection limits of 28.384 pg/L and 28.125 ng/L, respectively. The average intra-assay coefficients of variation (intra-assay CV) for the used enzyme immunoassay tests (ELISA) were <8%. All samples were analyzed in duplicate in a single assay to avoid inter-assay variability.

### 4.4. Statistical Analysis

Initial Correlation Analysis was performed with the use of Spearman’s rank correlation coefficients (ρ/rho) to assess bivariate relationships between individual eicosanoids, IL-1β, and IL1-Ra levels. The skewness and kurtosis values showed moderate non-normality, so this test was applied. Statistical significance was set at *p* < 0.05. Multivariate analysis with the use of principal component analysis (PCA) was performed to address multicollinearity among fatty acids. The data was standardized prior to analysis. Components were retained based on eigenvalues greater than 1, and Varimax rotation was applied to enhance interpretability of the components. Partial correlation analysis was performed to evaluate the independence of relationships and confounding assessment. The relationships between eicosanoids, IL-1β, and lL1-Ra were reassessed while controlling for other eicosanoids. Changes in correlation coefficients exceeding 0.1 were considered indicative of potential confounding. Multiple linear regression was conducted using the PCA components as predictors. Model assumptions were verified. R-squared and adjusted R-squared values were calculated to assess the proportion of variance explained by the model. F-statistics and corresponding *p*-values were computed to evaluate model significance. Individual component contributions were assessed using t-statistics and their associated *p*-values. Principal Component Analysis was performed on standardized variables using median imputation for missing values. The analysis identified orthogonal components explaining maximum variance in the dataset. Component loadings were interpreted to understand variable relationships, with loadings > |0.3| considered meaningful. Variance explained by each component was calculated, and cumulative variance was used to determine the number of components to retain. Multiple testing correction was applied using the Benjamini–Hochberg False Discovery Rate (FDR) method to control the expected proportion of false discoveries among rejected hypotheses. The FDR-adjusted *p*-values (q-values) were calculated to maintain an overall false discovery rate of 5%, providing a less conservative approach than family-wise error rate corrections while controlling for multiple comparisons. Bonferroni correction was applied to control family-wise error rate by adjusting the significance threshold (α = 0.05/n, where n = number of comparisons). All statistical analyses were performed in Python (version 3.x) using the scipy.stats, sklearn, and stats models packages.

## Figures and Tables

**Table 1:** Demographic characteristics of subjects.

Parameter	Value
Age (years)	60.38 ± 11.69
Sex (Male)	33 (44.6%)
BMI (kg/m^2^)	28.75 ± 5.03
Hypertension	60 (82.2%)
Diabetes or IFG	34 (46.6%)
Current smoking	26 (35.6%)
Dyslipidemia	41 (56.2%)
Ischaemic heart disease	8 (11%)
Previous stroke	5 (6.8%)
CRP (mg/L)	2.79 ± 3.93
Total Cholesterol (mg/dL)	195.82 ± 52.86
LDL (mg/dL)	114.36 ± 44.83
HDL (mg/dL)	52.16 ± 15.23
Non-HDL (mg/dL)	143.06 ± 50.56
Triglycerides (mg/dL)	154.6 ± 75.78

**Table 2:** Unadjusted correlations between eicosanoids and IL-1β.

Eicosanoid (µg/mL)	Mean	n	SD	Spearman Correlation	*p*-Value
Resolvin E1	0.059114	73	0.091654	0.153809	0.197064
Prostaglandin E2	3.39386	72	4.261734	0.229074	0.054658
Resolvin D1	0.173401	72	0.255647	0.131424	0.274619
LTXA4 5S, 6R, 15R (Lipoxin A4 5S, 6R, 15R)	0.06695	25	0.046868	0.302867	0.141122
10S17R DiHDHA (protectin DX)	0.059298	58	0.06683	0.559357	<0.001
Maresin 1	0.031977	71	0.014459	0.114281	0.346182
Leukotriene B4	0.027423	71	0.012905	−0.21579	0.072793
18RS HEPE	0.108684	73	0.037517	0.038849	0.744184
13S HODE	0.033289	73	0.029106	−0.03504	0.770133
9S HODE	0.03399	73	0.027604	−0.06963	0.561119
15S HETE	0.295675	73	0.204213	0.016145	0.892926
17 HDHA	0.122553	73	0.085322	0.257562	<0.05
12S HETE	1.783897	73	1.137498	−0.00318	0.97871
5-oxo-ETE	0.193363	71	0.095486	0.266243	<0.05
5 HETE	0.025014	73	0.012951	0.092138	0.438165

**Table 3:** Unadjusted correlations between eicosanoids and IL1-Ra.

Eicosanoid (µg/mL)	Mean	n	SD	Spearman Correlation	*p*-Value
Resolvin E1	0.059114	73	0.091654	−0.06302	0.598982
Prostaglandin E2	3.39386	72	4.261734	−0.11198	0.352504
Resolvin D1	0.173401	72	0.255647	−0.0605	0.61623
LTXA4 5S, 6R, 15R (Lipoxin A4 5S, 6R, 15R)	0.06695	25	0.046868	−0.12118	0.56394
10S17R DiHDHA (protectin DX)	0.059298	58	0.06683	−0.57978	<0.001
Maresin 1	0.031977	71	0.014459	−0.04142	0.733536
Leukotriene B4	0.027423	71	0.012905	0.338177	<0.005
18RS HEPE	0.108684	73	0.037517	0.104043	0.381041
13S HODE	0.033289	73	0.029106	0.035021	0.770248
9S HODE	0.03399	73	0.027604	0.034812	0.771582
15S HETE	0.295675	73	0.204213	0.079416	0.507252
17 HDHA	0.122553	73	0.085322	−0.28791	<0.05
12S HETE	1.783897	73	1.137498	−0.05227	0.660537
5-oxo-ETE	0.193363	71	0.095486	−0.10192	0.401138
5 HETE	0.025014	73	0.012951	−0.09634	0.417453

## Data Availability

The datasets generated during and/or analyzed during the current study are not publicly available but are available from the corresponding author on reasonable request.
